# Laparoscopic left hemihepatectomy is suitable as a first step in pure laparoscopic major hepatectomy

**DOI:** 10.1002/ags3.12193

**Published:** 2018-07-13

**Authors:** Yasushi Hasegawa, Hiroyuki Nitta, Takeshi Takahara, Hirokatsu Katagiri, Shoji Kanno, Koki Otsuka, Akira Sasaki

**Affiliations:** ^1^ Department of Surgery Iwate Medical University School of Medicine Morioka City Iwate Japan

**Keywords:** laparoscopy, learning curve, liver resection, major hepatectomy

## Abstract

**Aim:**

As a procedure, major laparoscopic liver resection (LLR) remains in the exploration phase. Previous studies have assessed major LLR en bloc, including hepatectomies of varying complexities; however, the number of segments alone does not convey the complexity of a resection. This study aimed to assess operative outcomes of LLR procedures with more than one sectionectomy, and to identify the best procedure as a first step when learning to carry out major LLR in order to make LLR a safer, more widely used procedure.

**Methods:**

We carried out a retrospective review of the operative outcomes of 120 consecutive patients who underwent pure LLR with more than one sectionectomy. Operative outcomes were compared according to the complexity classification recently published, and the learning curve for each LLR procedure was assessed and compared.

**Results:**

Operative outcomes, including operative time, blood loss, and the comprehensive complication index, were significantly stratified according to complexity. There were significant differences in operative outcomes among the medium complexity procedures. The operative time for left hemihepatectomy was the shortest, and the amount of blood loss was the lowest among the medium complexity LLR. Operative times for left hemihepatectomy shortened significantly with time and experience (*r* = −0.639), and the slope of the learning curve was steeper than for right hemihepatectomy and right posterior sectionectomy.

**Conclusion:**

Left hemihepatectomy is suitable as a first step in pure laparoscopic major hepatectomy and, given its safety and rapid learning curve for surgeons, it could become the gold standard procedure.

## 1 INTRODUCTION

Laparoscopic liver resection (LLR) surgeries are still being developed, meaning they are not as widely used as open liver surgeries, despite their clinical benefits. These benefits include less blood loss, less pain and analgesic requirement, shorter hospital stays, and improved cosmetic results.^1^, ^2^, ^3^, ^4^, ^5^, ^6^, ^7^ International Consensus Conferences on LLR have been held to evaluate the status of laparoscopic liver surgery, and these conferences have provided recommendations to aid in the future development of this procedure.^8^, ^9^ During the second conference, the major type of LLR that was discussed was an innovative procedure still in the exploration phase with incompletely defined risks; therefore, extending the clinical indications for this LLR should be considered carefully.^9^

In several previous studies, various procedures were assessed and discussed, particularly the learning curve, en bloc as a major LLR.^3^, ^10^, ^11^, ^12^, ^13^, ^14^, ^15^ The complexity of each major hepatectomy procedure differs widely, and the number of segments alone does not convey the complexity of a resection. To this end, Lee et al^16^, ^17^ recently reported that the complexity of open liver resections should not be classified based on whether the resection is “major or minor,” but instead based on the extent of the liver resection.

There are no reports comparing the learning curve for each different type of major LLR procedure. Here, we aimed to investigate which of these major LLR procedures should be the first step for surgeons when starting to carry out major LLR.

## 2 PATIENTS AND METHODS

### 2.1 Data source and study population

The prospective database of patients treated with pure LLR with more than one sectionectomy at our institution was retrospectively reviewed. All patients provided informed consent for the procedure. This study was approved by our institutional review board.

We have been carrying out LLR procedures at our institution since May 1997, and approximately 600 patients have undergone LLR as of October 2017. At first, the laparoscopic peripheral wedge resection procedure was introduced, and the extent of resection was extended in a stepwise method. Laparoscopic left lateral sectionectomy (LLLS) was the first sectionectomy adopted in April 2003. Right hemihepatectomy was the first major LLR introduced in September 2009.

The clinical records of the consecutive 120 patients who underwent a pure LLR with more than one sectionectomy were reviewed to extract the following information for analysis: patient characteristics, tumor characteristics, operative procedures, and operative outcomes. First, the operative outcomes were compared according to Lee's complexity classification system for hepatectomy.^16^, ^17^ The classification divides open liver resection procedures into three complexity groups: low, medium, and high. In this system, a left lateral sectionectomy is classified as low complexity. Left hemihepatectomy, right hemihepatectomy, and posterior sectionectomy are considered medium complexity, whereas right anterior sectionectomy and central bisectionectomy are considered high complexity. Next, the outcomes of each LLR procedure classified as medium complexity were compared to evaluate the learning curve, and to explore which procedure was suitable as the first step in major LLR. This analysis was carried out after also excluding cases involving associated procedures (e.g. stoma closure, colectomy, or radiofrequency ablation) and/or multiple hepatectomies. In this study, an expert was defined as a surgeon who had experience in carrying out more than 60 LLR, either as a surgeon or as an assistant surgeon.^10^, ^11^ In the present study, three experts and six non‐experts carried out the LLR.

### 2.2 Definitions

Extent of liver resection was classified according to the Brisbane 2000 terminology.^18^ Postoperative morbidity and mortality were defined as any complication or death within 90 days, respectively. Complications were graded according to the Clavien‐Dindo classification system and were scored by the comprehensive complication index (CCI^®^).^19^, ^20^ CCI^®^ was calculated online by http://www.assessurgery.com. For example, a patient who had one grade IIIa complication was scored 26.2, and a patient who had two grade II complications was scored 29.6.

### 2.3 Surgical technique

Our basic surgical LLR techniques were carried out as previously described.^21^, ^22^ The Glissonian approach or individual hilar dissection for the hilar approach was chosen on a case‐by‐case basis. The liver parenchyma was transected along the demarcation line and the main hepatic vein. After April 2012, the intermittent Pringle maneuver was routinely used except for LLLS. Additionally, low central venous pressure, the reverse Trendelenburg position, low airway pressure, and low tidal volume contributed to less bleeding from the hepatic vein.^5^, ^23^, ^24^

### 2.4 Statistical analysis

Continuous data were expressed as median values with the associated interquartile ranges. Categorical data were expressed as counts, with the associated percentile values calculated. The Kruskal‐Wallis test was used to compare continuous data, and the chi‐squared test was used for categorical data. *P*‐value <0.05 was considered statistically significant. All statistical analyses were carried out using JMP statistical software (version 9.0.0; SAS Institute, Cary, NC, USA).

## 3 RESULTS

Patient and tumor characteristics are shown in Table 1. Rate of malignant lesions was 88.3%, median tumor size was 35 mm (24‐61 mm), and liver cirrhosis was observed in 15 patients (12.5%).

**Table 1:** Patient and tumor characteristics

Number of patients	120
Age (years), median (IQR)	65 (57‐73)
Gender (male), n (%)	72 (60.0)
BMI (kg/m^2^), median (IQR)	23.1 (20.5‐25.7)
Diagnosis (HCC/CRLM/other malignancy/benign), n (%)	51/42/13/14 (42.5/35.0/10.8/11.7)
Tumor size (mm), median (IQR)	35 (24‐61)
Multiple tumors, n (%)	32 (26.7)
Child‐Pugh grade B, n (%)	1 (0.8)
ICG‐R15 (%), median (IQR)	11 (7‐15)
HBV‐Ag positive, n (%)	9 (7.5)
HCV‐Ab positive, n (%)	15 (12.5)
Liver cirrhosis, n (%)	15 (12.5)

BMI, body mass index; CRLM, colorectal liver metastasis; HBV‐Ag, hepatitis B virus antigen; HCC, hepatocellular carcinoma; HCV‐Ab, hepatitis C virus antibody; ICG‐R15, indocyanine green; IQR, interquartile range.

Operative methods and outcomes are summarized in Table 2. Median operative time was 251 minutes (144‐334 minutes) and the amount of blood loss was 43 mL (13‐187 mL). Conversion to an open laparotomy procedure was observed in four cases (3.3%). These conversions were as a result of two bile duct repairs, one case of bleeding from the extrahepatic portal vein, and one case of severe adhesion as a result of previous surgeries. Rates of grade I and greater than grade IIIa postoperative complications according to the Clavien‐Dindo classification system were 43.3% and 11.7%, respectively.

**Table 2:** Operative methods and outcomes of laparoscopic liver resection with more than one sectionectomy

Operative procedure (Lat/Med/Ant/Post/Left/Right/Cent), n (%)	46/4/8/17/26/18/1 (38.3/3.3/6.7/14.1/21.7/15.0/0.8)
Multiple hepatectomies during a surgery, n (%)	12 (10.0)
Associated procedure, n (%)	26 (21.7)
Lee's complexity classification (Low/Medium/High), n (%)	46/61/9 (39.7/52.6/7.7)
Operative time (min), median (IQR)	251 (144‐334)
Blood loss (mL), median (IQR)	43 (13‐187)
Blood transfusion, n (%)	1 (0.8)
Conversion to open laparotomy, n (%)	4 (3.3)
Length of hospital stay (days), median (IQR)	10 (7‐14)
Readmission, n (%)	5 (4.2)
Complications, n (%)	52 (43.3)
Complications (grade ≥IIIa), n (%)	14 (11.7)
CCI^®^, median (IQR)	0 (0‐20.9)
CCI^®^ ≥26.2, n (%)	21 (17.5)
Mortality, n (%)	2 (1.7)

Ant, right anterior sectionectomy; CCI, comprehensive complication index; Cent, central bisectionectomy; IQR, interquartile range; Lat, left lateral sectionectomy; Left, left hemihepatectomy; Med, left medial sectionectomy; Post, right posterior sectionectomy; Right, right hemihepatectomy.

According to Lee's complexity classification, 46 patients (39.7%) were considered as low, 61 (52.6%) as medium, and nine (7.7%) as high complexity cases. Operative outcomes for the patients who underwent LLR without associated procedures according to Lee's complexity classification are shown in Table 3. Operative time, blood loss, duration of Pringle maneuver, length of hospital stay, and postoperative complications were significantly stratified by the three complexity groups.

**Table 3:** Operative outcomes according to Lee's complexity classification

	Low (n = 37)	Medium (n = 45)	High (n = 9)	*P*‐value
Operative time (min), median (IQR)	127 (105‐144)	302 (243‐381)	432 (294‐490)	<.0001
Blood loss (mL), median (IQR)	13 (5‐24)	153 (40‐307)	211 (69‐313)	<.0001
Pringle maneuver (min), median (IQR)	0 (0)	54 (45‐74)	140 (98‐161)	<.0001
Conversion to open laparotomy, n (%)	1 (2.7)	2 (4.4)	0 (0.0)	.766
LOS (days), median (IQR)	7 (5‐9)	11 (9‐15)	12 (11‐19)	<.0001
Readmission, n (%)	0 (0)	3 (6.7)	1 (11.1)	.200
Complication, n (%)	8 (21.6)	22 (48.9)	7 (77.8)	.003
Complication (grade ≥IIIa), n (%)	0 (0)	5 (11.1)	2 (22.2)	.039
CCI^®^, median (IQR)	0 (0‐0)	0 (0‐8.7)	0 (0‐23.6)	.045
CCI^®^ ≥26.2, n (%)	0 (0)	10 (22.2)	2 (22.2)	.009
Mortality, n (%)	0 (0)	0 (0)	1 (11.1)	.010

CCI, comprehensive complication index; IQR, interquartile range; LOS, length of hospital stay.

**Figure 1:**
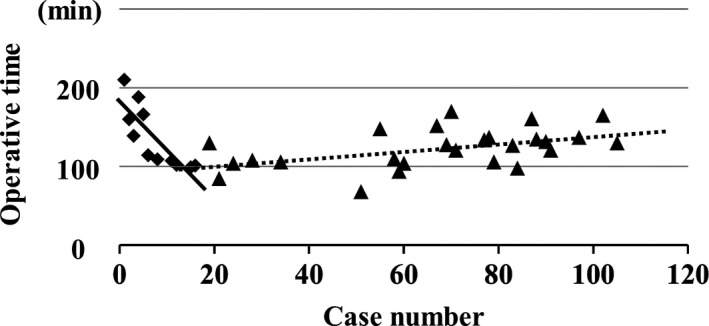
Operative times for laparoscopic left lateral sectionectomy. Solid line and ◆ show the linear approximation equation of the former 14 cases (*y* = 182 − 6.16*x*, where *r* = −0.822), and the dotted line and ▲ show that of the latter cases (*y* = 91.4 + 0.48*x*, where *r* = 0.486)

Low complexity procedures consisted of left lateral sectionectomies. The first 14 LLLS were carried out by expert surgeons only, and the following 32 cases were carried out by experts or non‐expert surgeons. Operative time of the early cases shortened rapidly, and the approximation formula was as follows: *y* = 182 − 6.16*x*, where *r* = −0.822. In contrast, that of the latter cases almost plateaued, with a formula of *y* = 91.4 + 0.48*x*, where *r* = 0.486 (Figure 1). These findings suggested that LLLS rapidly standardized and became the gold standard operation.

**Figure 2:**
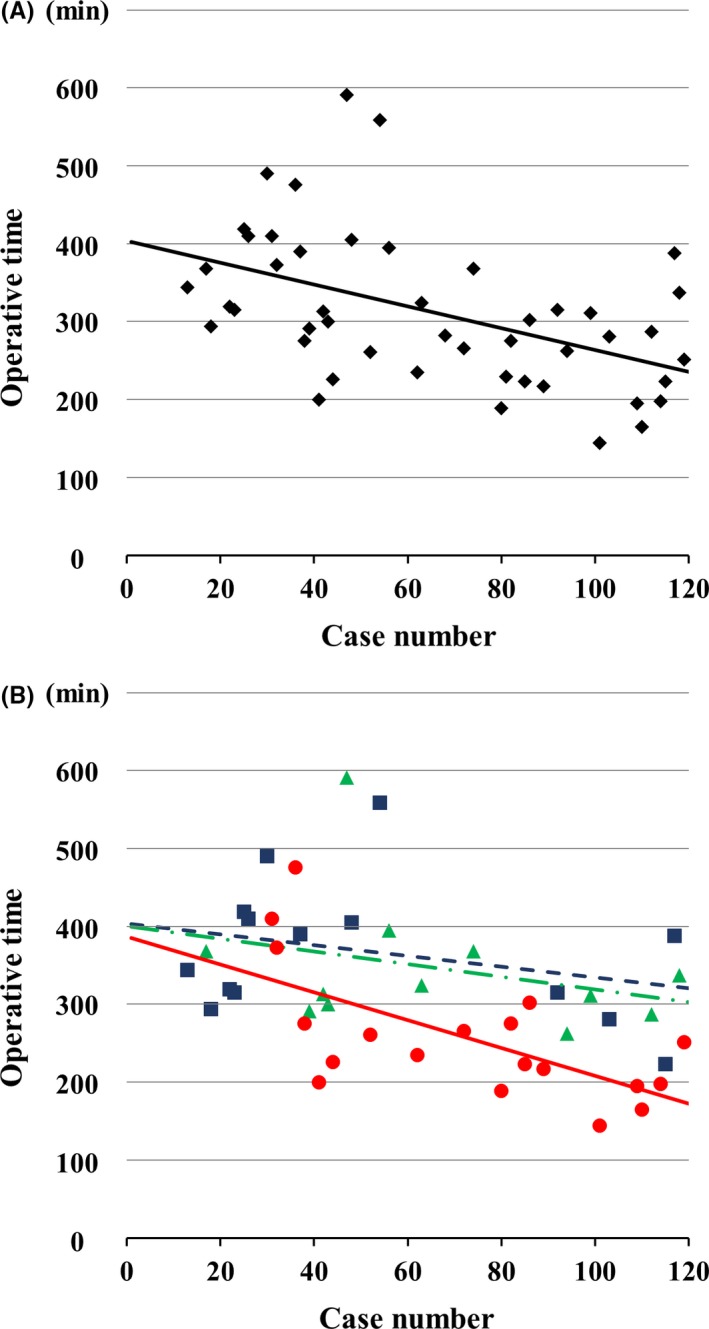
A, Operative times for all middle complexity hepatectomies. The linear approximate equation is *y* = 404 − 1.40*x* (*r* = −0.479). B, Laparoscopic left hemihepatectomy (red, solid line and ●), right hemihepatectomy (blue, dashed line and ■), and right posterior sectionectomy (green, dot‐dash line and ▲) are shown. The linear approximation formulae are as follows: left hemihepatectomy, *y* = 387 − 1.79*x* (*r* = −0.639); right hemihepatectomy, *y* = 404 − 0.70*x* (*r* = −0.302); right posterior sectionectomy, *y* = 400 − 0.81*x* (*r* = −0.305)

**Figure 3:**
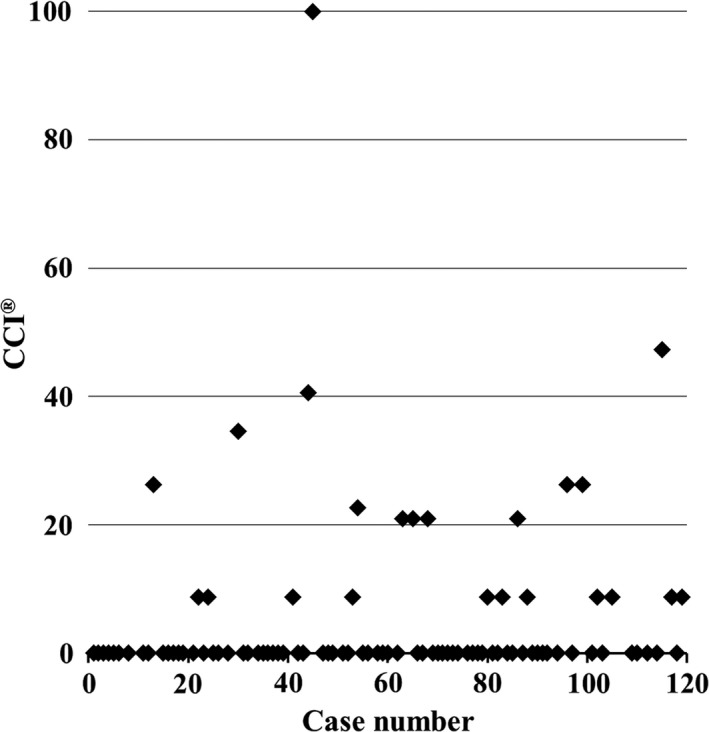
Comprehensive complication index (CCI ^®^) in a time series graph

In the medium complexity group, there were three different LLR procedures, which included left hemihepatectomies without caudate resection (n = 19), right hemihepatectomies (n = 14), and posterior sectionectomies (n = 12). The operative outcomes for these three procedures are shown in Table 4. Among them, left hemihepatectomies had the shortest operative time, the shortest Pringle maneuver time, and the least blood loss, whereas right hemihepatectomies had the highest rate of complications. The operative times in chronological order are shown in Figure 2A. These operative times gradually, but significantly, shortened over time through trial and error (*y* = 404 − 1.40*x*, where *r* = −0.479). Furthermore, the slope of the approximation formula for the left hemihepatectomy operative times was steeper than either that of right hemihepatectomy or right posterior sectionectomy (Figure 2B). The formulae for these operative times are as follows: left hemihepatectomy, *y* = 387 − 1.79*x*, where *r* = −0.639; right hemihepatectomy, *y* = 404 − 0.70*x*, where *r* = −0.302; and right posterior sectionectomy, *y* = 400 − 0.81*x*, where *r* = −0.305. The CCI^®^ of the time series graph is shown in Figure 3. It tended to be flat and of a low level.

**Table 4:** Operative outcomes according to the extent of hepatectomy in the medium complexity procedures

	Left (n = 19)	Right (n = 14)	Post (n = 12)	*P*‐value
Operative time (min), median (IQR)	235 (198‐275)	366 (310‐412)	319 (293‐368)	.001
Blood loss (mL), median (IQR)	38 (15‐164)	206 (137‐490)	202 (67‐299)	.009
Pringle maneuver (min), median (IQR)	45 (41‐51)	67 (51‐86)	75 (66‐102)	.001
Conversion to open laparotomy, n (%)	2 (10.5)	0 (0)	0 (0)	.239
LOS (days), median (IQR)	10 (8‐14)	12 (9‐26)	11 (9‐14)	.522
Readmission, n (%)	1 (5.3)	2 (14.3)	0 (0)	.329
Complication, n (%)	8 (42.1)	11 (78.6)	3 (25.0)	.018
Complication (grade ≥IIIa), n (%)	1 (5.3)	3 (21.4)	1 (8.3)	.323
CCI^®^, median (IQR)	0 (0‐8.7)	0 (0‐23.5)	0 (0‐0)	.290
CCI^®^ ≥26.2, n (%)	4 (21.1)	5 (35.7)	1 (8.3)	.243
Mortality, n (%)	0	0	0	1.000

CCI, comprehensive complication index; IQR, interquartile range; Left, left hemihepatectomy; LOS, length of hospital stay; Post, right posterior sectionectomy; Right, right hemihepatectomy.

## 4 DISCUSSION

As a procedure, LLR has a relatively short history; hence, it remains experimental. Brown and Geller^25^ reported that surgeons should begin with the laparoscopic peripheral wedge resection, and experience with anatomical LLLS should be acquired before any attempt at a major LLR is carried out. Hasegawa et al^11^ demonstrated that a major LLR could be safely introduced for a surgeon with an experience level of at least 60 minor LLR. Nomi et al^12^ reported that 45 major LLR were required before operating times were reduced. Thus, several studies have described the learning curve for major LLR en bloc; however, it is clear that the number of segments alone does not convey the complexity of a resection. It was important, therefore, to explore which type of major LLR procedure was suitable as a first step, which is what has been presented in the present study.

Lee et al^16^, ^17^ presented the first quantitative assessment of the perceived difficulty of a variety of open liver resections, and the complexity scores that were generated allowed for these resections to be separated into three categories of complexity. According to Lee's complexity model, LLR performed in our institution were well classified in terms of operative time, blood loss, and postoperative complications.

Laparoscopic left lateral sectionectomy has been acknowledged as the standard procedure.^22^, ^26^, ^27^, ^28^ In this study, the first 14 cases showed a rapid learning curve, that later plateaued, regardless of whether the procedures were carried out by not only experts but also by non‐expert surgeons. These results suggested that the LLLS procedure was well standardized, and the results aligned with the current view that LLLS should be the standard procedure. Additionally, its learning curve was steeper than that of medium‐complexity LLR.

Three procedures, categorized into the medium complexity category, were further compared. Laparoscopic left hemihepatectomy showed the shortest operative time, the least amount of blood loss, and a rapid learning curve. Furthermore, the duration of Pringle maneuver for left hemihepatectomy was approximately 30 minutes shorter compared to right hemihepatectomy and posterior sectionectomy. In our institution, the intermittent Pringle maneuver was routinely carried out during liver parenchymal transection, and not for hemostasis after bleeding; therefore, the results reflected shorter parenchymal transection times for left hemihepatectomy. These findings might indicate that pure laparoscopic left hemihepatectomy is suitable as a first step in pure laparoscopic major hepatectomy. This concept aligns well with the report by Takahara et al^7^ from the national clinical database in Japan, concluding that among the major LLR procedures, a left hemihepatectomy could be a good option for standard practice.

The CCI^®^ was developed to overcome an underestimation of the true overall postoperative morbidity.^19^, ^20^ In this study, the CCI^®^ was used to evaluate postoperative morbidity, and low values were obtained throughout our study period. These findings suggest that the learning curve did not influence morbidity. The reason for this could be that LLLS was adopted as the first LLR with more than one sectionectomy, which was a relatively easier procedure; therefore, there were no major complications. Additionally, it could be because major LLR was adopted only after adequate experience was acquired with minor LLR. The indications for hepatectomy were extended in a stepwise method to ensure patient safety.

In the present study, the surgeries of two patients in the left hemihepatectomy group were converted to laparoscopic‐assisted procedures. The first patient had massive bleeding from the portal vein caused by the Pringle maneuver itself, and mini‐laparotomy was required to suture and achieve hemostasis. The second patient had bile leakage following a left hepatic duct stapling failure caused by severe inflammation of the bile duct. These complications could have actually occurred in any type of hepatectomy. Overall, the rate of conversion to open laparotomy was very low, so it would not be a factor contributing to the learning curve in this study.

The greatest advantage of LLR compared to open hepatectomy is that less bleeding from the hepatic vein occurs as a result of pneumoperitoneum.^5^, ^23^, ^24^ However, the disadvantages involve movement restrictions and the disorientation that can be caused by the limited surgical view.^29^ Our transection method for left hemihepatectomy was progressed along the demarcation line and the middle hepatic vein in the craniocaudal direction. These procedures are suitable for LLR because less bleeding occurs from the hepatic vein, and contribute to overcoming the disadvantages of LLR. Moreover, a left hemihepatectomy is theoretically a more acceptable procedure compared to a right hemihepatectomy or a right posterior sectionectomy. First, left hemihepatectomy has a lower potential risk of bleeding from the inferior vena cava (IVC), because dissection between the IVC and the liver is not needed. Second, the parenchymal transection area of left hemihepatectomy is smaller compared to right hemihepatectomy or posterior sectionectomy. Finally, parenchymal transection along the Cantlie line is easier than that of the right intersectional plane because of both the lower risk of disorientation and the lower bleeding risk because of the lower hepatic venous pressure.

Regarding the operative techniques, there are two methods of hilar dissection, the Glissonian approach and individual dissection. In the present study, the operative outcomes of the two methods could not be compared because of differences in patient backgrounds. Both methods are generally acceptable, and neither is superior in terms of safety and reproducibility. Therefore, the operative method is left to the surgeon's discretion and preference.^30^ Spiegel's lobe resection is another concern. Spiegel's lobe resection requires dissection from the IVC, and division of the Glissonian vessels in Spiegel's lobe and the larger parenchymal transection area. Therefore, the risk of bleeding and bile leakage might increase and prolong the operative time.

This study had several limitations. First, this report includes only the experiences gathered from a single, specialized institution. Additionally, the number of cases in our study group was limited and, consequently, the learning curve for the LLR apart from four procedures could not be evaluated. Therefore, validations, multicenter comparison, learning curve analyses, or comparisons among surgeons with varied experiences are required using independent data sets. Despite these limitations, we believe that a pure laparoscopic left hemihepatectomy without Spiegel's lobe resection is suitable as a first step for pure laparoscopic major hepatectomy and, given its safety and rapid learning curve for surgeons, could become the gold standard procedure.

## DISCLOSURE

The protocol for this research project has been approved by a suitably constituted ethics committee of the institution, and it conforms to the provisions of the Declaration of Helsinki. Committee of Iwate Medical University School of Medicine, Approval No. H29‐131. All informed consent was obtained from the subjects.

Conflicts of Interest: Authors declare no conflicts of interest for this article.

Author Contribution: All authors were involved in the conception, design, interpretation of data, surgery, and acquisition of data. YH and TT analysed the data.
